# Recent Overviews in Functional Polymer Composites for Biomedical Applications

**DOI:** 10.3390/polym10070739

**Published:** 2018-07-04

**Authors:** Moustafa M. Zagho, Essraa A. Hussein, Ahmed A. Elzatahry

**Affiliations:** Materials Science and Technology Program, College of Arts and Sciences, Qatar University, Doha P.O. Box 2713, Qatar; mmsalah@qu.edu.qa (M.M.Z.); eh1604846@student.qu.edu.qa or ekhalil@qu.edu.qa (E.A.H.)

**Keywords:** polymer composites, biomedical, tissue engineering, dental resin-based composites, wound dressing

## Abstract

Composite materials are considered as an essential part of our daily life due to their outstanding properties and diverse applications. Polymer composites are a widespread class of composites, characterized by low cost, facile processing methods, and varied applications ranging from daily-use issues to highly complicated electronics and advanced medical combinations. In this review, we focus on the most important fabrication techniques for bioapplied polymer composites such as electrospinning, melt-extrusion, solution mixing, and latex technology, as well as in situ methods. Additionally, significant and recent advances in biomedical applications are spotlighted, such as tissue engineering (including bone, blood vessels, oral tissues, and skin), dental resin-based composites, and wound dressing.

## 1. Introduction

A composite is a material structure that consists of two or more macroscopically identifiable components that work together to attain a better property. Generally, composites consist of two phases: a dispersed phase and a matrix phase. They offer novel characteristics which, importantly, are different from those of their components [1]. Researchers have paid their attention to composite materials because of their promising properties such as corrosion resistance, low weight, and high fatigue strength. Composites are synthesized in such a way as to attain homogenous and controlled distribution of the materials. Polymer composites are widely applied in numerous biomedical applications such as dental, tissue engineering, and regenerative medicine applications [2,3].

A biocomposite material is a combination of matrices such as polymer and reinforced natural fibers. These composites mimic the morphology of the living materials with excellent biocompatibility. The polymer matrix protects the fibers from mechanical damage and environmental degradation. Moreover, biofibers are natural fibers produced from biological origins such as wood, crops, and regenerated cellulose. Biocomposites enhance the safety in their production [4]. They are environmentally friendly, lighter in weight, cheap, completely recyclable in specific cases, and renewable sources of composites [4]. Biocomposites are divided into wood and non-wood fibers, which all contain lignin and cellulose. Wood fibers have a low degree of cellulose crystallinity and include hardwood and softwood fibers. The non-wood fibers are applied in the industry because of their good mechanical and physical properties. In contrast, they might swell because of containing OH groups that can attract water molecules.

The purpose of this review is to spotlight benefits of employing polymer composites in recent biomedical applications connected to different fabrication technologies of polymer composites. 

## 2. Composites Classification

**Figure 1:**
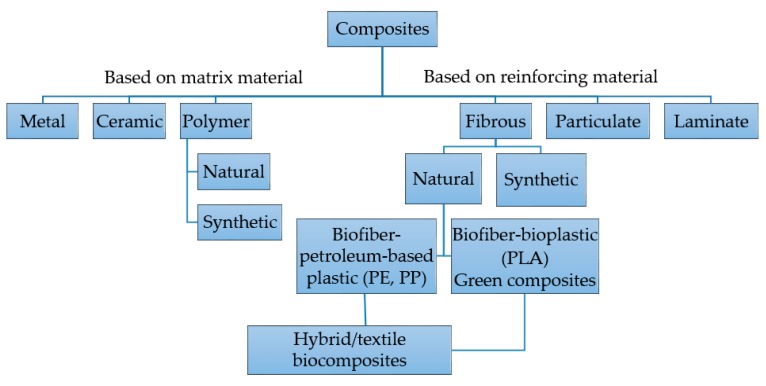
Classification of composites (polyethylene (PE), polypropylene (PP), and polylactic acid (PLA)). Reproduced with permission from [1]. John Wiley and Sons, 2012.

Based on the concept of the matrix phase, several reports have reported the classification of composites into metal matrix composites (MMCs), ceramic matrix composites (CMCs), and polymer matrix composites (PMCs) (see Figure 1) [1,5]. According to the basis of reinforcement, they are classified into fibrous, particulate, and laminate composites. Fibrous ones are classified into synthetic fiber and natural biofibers. Biofiber composites can be divided on the basis of degradable and non-biodegradable matrixes [1,6]. Biobased composites made from biodegradable polymers and natural biofibers are referred to as green composites. These are again classified as textile and hybrid composites. In addition, hybrid ones consist of a mixture of two or more kinds of fibers. Here, we will illustrate in detail the polymer matrix composites, including fabrication techniques and biomedical applications.

PMCs are widely applied and broadly categorized owing to polymer types and availability, their simple fabrication processes, and low cost. Polymers such as polyacrylic, polylactic acid (PLA), and polyglycolide (PGA)/PLA copolymers are used for biomedical applications [7]. Fibers are the most commonly used reinforcement structures [8]. Some pure polymers without a reinforced component displayed poor tensile characters such as impact resistance, modulus, and tensile strength. More interestingly, PMC preparation is widely requested, particularly from reinforced polymers with fibers, which provided desirable behaviors such as low cost, excellent corrosion, abrasion, and impact resistance as well as high breakage resistance, specific strength, and stiffness. 

## 3. Fabrication Technology of Polymer Composites

Various processes are designed to process functional polymer composites for a wide range of applications, including biomedical fields. These techniques include electrospinning, melt-extrusion, solution mixing, latex technology, and in situ methods [9,10], which are concisely discussed in the next sections.

### 3.1. Electrospinning

There have been great efforts devoted to the electrospinning technique, owing to its ability to process fibers with diameter ranging from 2 nm to many micrometers by using a high-strength electric field. This technique is the one most applied to process nanofibers with a larger surface area than those prepared by common spinning techniques. The basic electrospinning system is composed of a spinneret, high-voltage power supply, and grounded collector [11,12]. The two standard types of electrospinning are vertical and horizontal sets [11,12]. In this approach, the polymeric solution is accelerated towards the oppositely charged collector. At a specific direct current (DC) voltage, the electrical repulsion forces surpass the surface tension of the polymeric solution. Then, an electrified jet will be emitted from the Tylor cone tip and the solvent will be evaporated, producing the fibers.

### 3.2. Melt-Extrusion

The extrusion technique is the most common method whereby to fabricate polymer composites. This technique depends on using a twin-screw extruder to mix polymer and fillers at a specific temperature for a certain period of time [12]. After extrusion, polymer composites will be shaped using compression molding under certain conditions for the final product. The mechanical and thermal characteristics of the prepared composites can be controlled by varying the filler content. For instance, polylactic acid (PLA) pellets and 5 wt % ceramic powder were blended in a microcompounder with two conical corotating screws of small capacity of 5 cm^3^, under a flow of nitrogen, to process ceramic/PLA bioresorbable composites [13]. A set of different screw rotation speeds (100, 150, and 200 rpm), temperature (200 and 205 °C), and residence times (1–4 min) were used. Moreover, Majeed et al. [14] prepared nanocomposites having 3 wt % nanotubes (carbon, titania, and halloysite) and 97 wt % low-density polyethylene (LDPE) using a Brabender Plastograph EC batch mixer operating for 7 min at 180 °C. Maleic anhydride grafted polyethylene (MAPE) was incorporated as a compatibilizing material because of the incompatibility between LDPE galleries and the nanotubes. To fabricate thin films, the mixed lumps were then compression-molded for 5 min at 200 MPa and 180 °C.

### 3.3. Solution Mixing 

Polymer composites can be processed using a solution technique, where the polymer is dissolved first at a specific temperature in a certain solvent, followed by homogeneous distribution of fillers such as montmorillonite clays into the polymer solution [12]. Constant stirring at a certain temperature for a fixed period of time is achieved, and then the resultant is dried in a mold with specified dimensions at a certain temperature for mechanical measurements [15,16]. As a case in point, Al-Marri et al. [17] synthesized a 10% poly(vinyl alcohol) (PVA) solution in water at 70 °C. The PVA/Cloisite^®^ 20A composites were fabricated by stirring Cloisite^®^ 20A suspensions with the PVA solution for 30 min at 70 °C. The mixture was then poured into a square aluminum mold (12 × 12 cm) and dried at 25 °C. The resulted films were then dried under vacuum at 60 °C. In addition, PLA pellets were first dissolved in chloroform to produce a 10% PLA solution by stirring for 2 h at 60 °C [13]. Afterward, ceramic powder was then incorporated into the PLA solution with stirring for 30 min. Drying at ambient temperature and further drying under vacuum for 2 days were used to evaporate the solvent.

### 3.4. Latex Technology

This technique has been designed to incorporate conductive fillers into a polymer network to produce conductive polymer composites. The latex process has many advantages such as process upscaling, easy processing, and homogeneously dispersed fillers in the polymer network [18]. Nanofillers can be incorporated directly into a highly viscous polymer network. Moreover, the 3D framework of filler particles can be formed successfully in polymer galleries by using this technology [18,19]. This process includes three steps: the processing of a colloidal dispersion of the nanofiller, mixing with a polymer latex, and lyophilization (drying) of the colloidal mixture [18]. This technique is used to produce carbon nanotube and graphene polymer composites. Carbon nanotube [20] and graphene [21] polymer composites can be used in different biomedical applications. 

### 3.5. In Situ Method

Fillers such as graphene or modified graphene can swell before incorporation into the matrix [22]. The filler is first swollen in the liquid monomer in the in situ polymerization process. Afterward, an initiator is incorporated and radiation or heat is used to initiate the polymerization step [22]. Carbon nanotube polymer composites can be processed by using an in situ polymerization method. This method can be applied to process polymer composites with high nanotube contents, which can be diluted by other methods [23]. 

## 4. Biomedical Applications of Polymer Composites 

Polymer composites have been explored in many applications, such as electrical and energy storage applications. Due to its superb characteristics such as high energy density, low cost, structural diversity, and design flexibility, the conducting polymer matrix has been widely applied as supercapacitors, electrochemical sensors, and in lithium-ion batteries [24,25,26,27]. The conducting polymers have been integrated with carbon nanomaterials such as carbon nanotubes (CNTs), graphene oxide (GO), and reduced graphene oxide (rGO) to enhance cycling stability, electrical conductivity, and provide high surface area [28,29,30,31]. Similarly, metal oxides or hydroxides could be composited with a polymer matrix to increase capacitance and provide better cyclic stabilities [32,33,34]. In addition, such conductive reinforcement materials (i.e., CNTs, graphite, carbon fibers, and metals), when added to a polymer matrix, resulted in polymer composites with improved thermal conductivity. Consequently, they are suitable to be applied as electronic package materials [35,36]. Furthermore, polymer composites are considered to be promising materials for biomedical applications [2,3].

It is worth addressing that the most remarkable advances in applications of polymer composites are the biomedical ones, including tissue engineering and wound healing. In addition, polymer-based nanoagents are the most addressed organic-based photothermal carriers for cancer therapy [37]. There are still critical challenges in the development of functional polymer composites. Consequently, there is a developing attention for the improvement of stable, efficient, and versatile processing techniques. Many growing industries are aiming to fulfill these requirements by offering novel, reliable, and environmentally friendly polymer composites. It is noteworthy to mention that polymer-based nanofibrous materials are used in many biomedical applications, such as tissue engineering, including of bones, blood vessels, and oral tissues and wound dressing [9]. 

### 4.1. Tissue Engineering

The concept of tissue engineering aims to regenerate damaged tissues with the support of scaffold biomaterials that act as a template to support the growth of new cells [38]. Biocompatible scaffolds based on the natural extracellular matrix (ECM) are cell- and tissue-specific materials that aid the fast integration of tissues [39].

Human cells can attach and organize well around polymer composites with dimensions smaller than those of the cells [40,41]. Polymer composites display a significant morphology which facilitates cells to migrate, grow, and seed. The improvement of polymer composites for cell proliferation and adhesion is important for organ and tissue reconstruction. In this concept, 3D biocompatible composites are required for cell growth for tissue repair and replacement. In general, polymer-based composites are promising templates to mimic the native morphology [40]. To illustrate this, Buchko et al. [42] processed porous biocompatible protein polymer thin films for use in implantable devices. Additionally, elastin-mimetic peptide polymers have been prepared and electrospun by Huang et al. [43].

#### 4.1.1. Bone

The bone of the skeletal system is considered as a morphological composite, consisting of collagen fibers with hydroxyapatite (HA) nanocrystals deposited along the collagen fibers [44]. Bone also consists of other materials such as bone cells, blood vessels, and mucopolysaccharides. The collagen fibers exhibit low elastic modulus and they are aligned in bone along the main stress direction. The HA mineral covers 70% of the dry bone weight and attributes to the bone stiffness. It is noteworthy to mention that bone can adapt and remodel itself in response to the applied mechanical surrounding, which is well known as Wolff’s law [7]. Bones are normally feeble with regard to shear and tension, mainly along the longitudinal plane. Fracture is one of the most common bone disorders that needs special medical care. There are different kinds of fractures, depending on the location, structure, orientation, and crack volume. Proper implant systems and surgical procedures must be controlled to attain the required biomechanical fixation and to bypass any extra tissue devascularization and trauma at the fracture point [45]. Moreover, the fracture healing process depends on the patient’s activities, as they control the stability of mechanical performance at the fracture site. All implants are temporarily sited inside the patient’s body and may be eliminated from the body after fracture healing. 

Scientists have been paying special effort to tissue engineering to improve biodegradable bone graft substitutes. Different degradable polymers, either synthetic or natural, have been used as scaffold systems for bone tissue engineering, such as chitosan [46,47], poly(propylene fumarate) [48], and polyesters, including the copolymer poly(lactide-co-glycolide) (PLAGA) [49,50]. For instance, PLAGA/calcium phosphate composite microsphere-based scaffolds were prepared by Khan et al. via in situ formation of calcium phosphate within forming microspheres [51]. The fabricated materials were incubated in simulated body fluid (SBF) for 8 weeks. It was hypothesized that a 11–14% drop in the composite scaffold molecular weight would occur, compared to a 20% drop in polymeric scaffolds over 8 weeks. In addition, SBF pH and composite scaffold mass declined first and then increased after two weeks, assumed to be a result of the chemistry between calcium phosphate dissolution and pH variations. The free calcium ion content of SBF containing composite scaffolds surged from 20 to 40% within 4 h of incubation and then declined to 40%, contributing to the burst release of calcium ions followed by reprecipitation on the scaffold surface. Moreover, these composite scaffolds initiated calcium phosphate reprecipitation for an bone/implant integration approach.

Polylactic acid (PLA) exhibits poor tensile characteristics for weight-bearing fixation systems. Hence, reinforcements including glass, ceramics, and alloys have been blended with PLA chains to enhance the mechanical performance [52,53]. Polymer composites containing phosphate glass fiber (PGF) displayed good biological and mechanical characteristics for repairing bone fractures [54]. For the approach of the design of bone plates applied in long-bone fractures, Mehboob et al. [55] synthesized unidirectional bioglass fibers (BGF) (13–93)/PLA composites treated with air plasma. Different doses of atmospheric air plasma exposure (30, 60, 90, and 120 s) were used on the surface of BGF to develop the interaction properties with the PLA galleries. The fatigue life of 30 s plasma-treated blends achieved 1 million cycles by using actual content conditions of 10–20% body weight. After 30 s plasma exposure, it was found that the interlaminar shear strength, flexural strength, and tensile strength were surged by 33%, 13.5%, and 31%, respectively. Also, the superior interaction between PLA network and fibers was evidenced by the failure shifting to the PLA. More interestingly, bone-like calcium phosphate layers were deposited on the surface of the decomposed blends, which is required for bone healing processes.

In addition, various biometallic materials have been remarkably applied in internal fixation processes due to their strength and toughness [56]. The desired implant should have the ability to degrade spontaneously inside the body to bypass its removal and stress shielding. For example, low-molecular-weight PLA was reinforced with various Mg rods by Butt et al. [57] to fabricate biodegradable PLA composite rods using a plastic injection molding (PIM) technique for bone fracture fixation. The authors selected magnesium fluoride (MgF_2_) in their experiments to strengthen the fabricated rods. In addition, hydrofluoric acid (HF) was used to produce a porous MgF_2_ ceramic layer on the Mg rod surface to enhance the interaction between the internal Mg rod and PLA network. It was realized that PLA-clad Mg composites with an intermediate coating displayed much better bending strength, tensile strength, and corrosion resistance in simulated body fluid (SBF) solution compared to PLA-clad Mg composites without coating. In contrast, the bending and tensile strength were diminished clearly with the presence of the intermediate coating, owing to the corrosion of the MgO porous interface.

**Figure 2:**
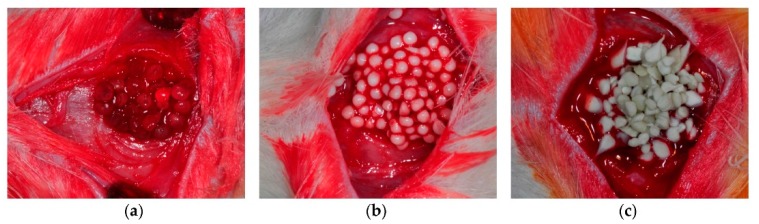
(**a**) Alginate; (**b**) HA/alginate; and (c) SF/HA/alginate beads were grafted into rat calvarial defects (silk fibroin (SF) and hydroxyapatite (HA)). Reproduced with permission from [61]. MDPI, 2017.

One of the most preferred types of material for repairing bone defects is the osteoconductive materials (such as HA [58]), which could simplify the growth of osteoprogenitor cells [59]. Recently, PLA/ethyl cellulose (EC)/HA composite scaffolds have been investigated as weight-bearing substitutes [60]. The mechanical performance of these scaffolds was improved by combining the particulate leaching, high content solvent casting, and room temperature compression molding methods. The mechanical and hydrophilicity characteristics of the composites were improved after incorporating HA and modifying the scaffold surface by sodium trimetaphosphate (STMP). The results revealed the PLA/EC/HA composites at 20 wt % HA content provided an excellent porous morphology and ideal mechanical behavior. The weight loss, compressive yield strength, contact angle, and porosity after 8 weeks were 4.77 ± 0.32%, 1.57 ± 0.09 MPa, 45.13 ± 2.40°, and 84.28 ± 7.04%, respectively. During hydrolysis, the scaffolds exhibited superior dimensional stability and porous morphology as well. In addition, alginate (AL)/HA/silk fibroin (SF) composites were fabricated by Jo et al. as bone tissue scaffolds for rat calvarial defects [61]. Figure 2 represents the calvarial defects grafted with AL, AL/HA, and AL/HA/SF beads. To fulfill the criteria for a unique osteoconductive candidate for bone defect repair, polypropylene carbonate (PPC)/poly(d-lactic acid) (PDLA)/β-tricalcium phosphate (β-TCP) (PDT) malleable composites were designed by Chang et al. [62]. The tissue biodegradation, compatibility, and osteoconductivity of the composites were evaluated at up to 12 weeks after incorporation in rabbit femur bone defects. The bone defect was regenerated with phosphate-buffered saline (PBS) and PPC/PDLA/β-TCP (PDT) scaffold in six rabbits, while it was left un-regenerated in four rabbits. The findings revealed that the PPC/PDLA/TCP scaffold with a weight ratio of 90/8/2 was a biodegradable, biocompatible, osteoconductive, and malleable biomaterial for bone defect repair.

#### 4.1.2. Blood Vessels

The repair of blood vessels is a challenging subject to researchers because most of the artificial scaffolds are not desirable for application, which leads to an increase of the time needed for patient recovery [63]. One of the main biomedical tasks for patients who require peripheral vascular bypass surgery and coronary artery is the vascular regeneration [64]. It is worth mentioning that tissue engineering vascular grafts, which combine synthetic vascular grafts with patient’s cells to replace and repair the injured vessels, are used as one of the most promising potential templates for biomedical applications at present [64,65,66]. To mimic the function and morphology of the extracellular matrix, electrospinning has been widely applied as a simple technique to fabricate 2D and 3D fibers using natural and artificial polymers [67]. This technique was widely used to process tubular scaffolds of various diameters and lengths for vascular grafts [68,69,70]. Electrospun tubular scaffolds with small diameters were processed from various synthetic polymers with vascular proteins [69,71]. 

**Figure 3:**
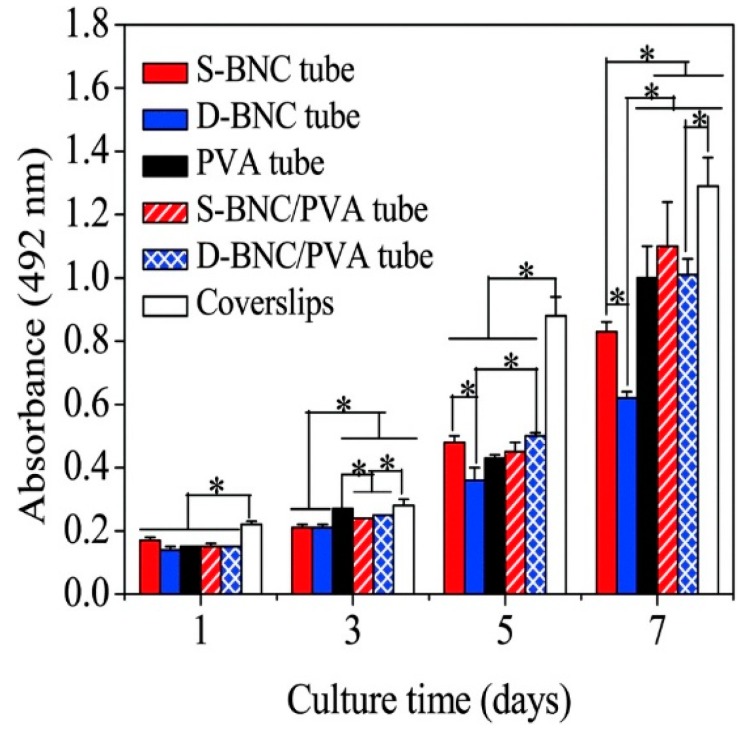
Proliferation of pig iliac endothelium cells (PIECs) on coverslips, bacterial nanocellulose (BNC) tubes, BNC/poly(vinyl alcohol) (PVA) composite tubes and PVA tubes at 1, 3, 5, and 7 days after cell seeding. Significant variance between groups is shown by asterisks (* *p* < 0.05). The tubes processed from the first bioreactor which was assembled with an about 60 mm silicone tube (inner diameter × external diameter: 2 × 3 mm) and a glass tube (8 × 10 mm), were defined as S-BNC tubes. The other bioreactor was consisted of two silicone tubes with various calibers (2 × 3 mm, 8 × 9 mm), were defined as D-BNC tubes. Reproduced with permission from [74]. Royal Society of Chemistry, 2015.

Bacterial nanocellulose (BNC), structured by repeated β-1,4 linked D-glucose dimers, has been reported to provide some novel properties, such as an ultrafine nanofiber matrix; high water holding capacity, crystallinity, and chemical purity; and excellent biocompatibility and wet tensile strength [72,73]. For instance, Tang et al. [74] evaluated BNC composite tubes doped with PVA as artificial blood vessels. The neat BNC tubes were considered to be an inadequate model for vascular grafts due to water leakage and poor suture retention. The water permeability and tensile characteristics were enhanced after blending BNC tubes with PVA. The mechanical properties of the fabricated composites were controlled by the BNC tube content. Two tubular bioreactors were employed for the processing of BNC tubes. The tubes produced from the first bioreactor which was assembled with an about 60 mm silicone tube (inner diameter × external diameter: 2 × 3 mm) and a glass tube (8 × 10 mm), were defined as S-BNC tubes. The other bioreactor was consisted of two silicone tubes with various calibers (2 × 3 mm, 8 × 9 mm), were defined as D-BNC tubes. The D-BNC/PVA composite exhibited a higher tensile strength when compared to S-BNC/PVA composite. This criterion is favored for veins and artery transplantation. Figure 3 describes the proliferation of pig iliac endothelium cells (PIECs) on coverslips, BNC tubes, BNC/PVA composite tubes, and PVA tubes at 1, 3, 5, and 7 days after cell seeding.

Another key point is that the composites of natural biopolymers such as chitosan, gelatin, collagen, alginate, and hyaluronic acid have been applied as implants and wound dressings as well as surgical sutures for decades [75,76]. Recently, to mimic the tensile and structural characters of biological blood vessels, macroporous blood vessels were developed by Badhe et al. through fabricating chitosan/gelatin bilayered composites [77]. On one hand, the macroporous layers displayed a large surface area for cell proliferation and adhesion. On the other hand, the outer nonporous layer offered higher elasticity, flexibility, and cell protection. The biodegradation of the macroporous layer with time was confirmed by fibroblast cell proliferation on the fabricated scaffolds. The viscoelastic nature (elastic (G′) and viscous (G′′) moduli) of the fabricated chitosan–gelatin hydrogel was measured at 37 °C. The experiments showed that the scaffolds had pore diameters between 100 and 230 µm, a porosity of 82%, elongation at fracture of 112.5 ± 13%, tensile strength of 95.81 ± 11 kPa, proliferated fibroblasts over 20 days, and 50% in vitro biodegradation after 16 days.

Owing to their low degradability and excellent mechanical characteristics, synthetic polymers such as poly(lactic-co-glycolic) acid (PLGA), PLA, and polycaprolactone (PCL) have been considered as promising scaffolds for constructing cardiac tissue [78]. Liu et al. [79] represented a platform for creating myocardia and for achieving in vitro cardiomyocyte cultures for drug screening by processing novel PLA/chitosan nanofibrous scaffolds. The scaffolds with random and aligned nanofibers were realized to promote the viability and cardiomyocyte attachment. On the one hand, aligned nanofibers enabled the construction of cardiac tissue through developing cardiomyocyte growth along their longitudinal axis. The processed fibers of PLA/chitosan (7:1) remarkably prepared the extracellular matrix as well as promoting the cell–scaffold bindings. Surface bioactivity plays a vital role in cell proliferation and adhesion. The blending of PCL with gelatin is an interesting concept whereby to overcome their individual limitations, in addition to being a promising approach for tissue engineering applications such as in muscles, skin, nerves, and teeth [80,81,82,83,84,85]. To demonstrate this, Jiang et al. blended PCL with gelatin to process composite fibers with improved cell–matrix interactions as blood vessel scaffolds [86]. The surface wettability of PCL fibers was remarkably modified from hydrophobic to hydrophilic after gelatin incorporation. The fiber diameters increased from 1.01 ± 0.51 µm to 1.61 ± 0.46 µm with increase of the gelatin content. The crystallization and thermal resistance of the scaffolds were significantly affected by the presence of gelatin. The high contents of gelatin inhibited crystallinity and decreased the tensile characteristics of the composites. On the other hand, scaffolds of 100% PCL and 70% PCL:30% gelatin (P7G3-C) were in agreement with the tensile characteristics of human coronary arteries. Cell proliferation and cytoskeleton staining studies revealed the developed bindings of mesenchymal stem cells with PCL/gelatin fibers. To sum up, the P7G3-C composite could be considered as a potential template for blood vessel tissue scaffolds.

#### 4.1.3. Skin

Being the first human body barrier against harmful antigens and pathogens, the skin is considered as one of the most vital body parts and the first immune system organ [87]. When skin is subjected to different stress conditions such as injury and serious burns, skin tissues will acquire secondary infections, necrosis, and damage which cannot be self-healed or repaired [87]. Consequently, developing biocompatible and biodegradable materials for skin regeneration has become a critical issue in the field of tissue engineering. The structural design of skin scaffolds and skin grafts should mimic the structure of the natural ECM [88].

Molding polymeric materials as 3D nanofibrous structures provides many advantages, such as a high surface area to volume ratio and excellent mechanical properties. Furthermore, polymeric nanofibers can be used as carriers for different drug molecules (e.g., antibiotic, antifungal, and anticancer drugs) [88,89]. Polymer-based hydrogels have been reported as one of the most convenient biomaterials used for this purpose [87]. Several types of hydrogels were fabricated for skin regeneration, being either natural, such as alginate, collagen, hyaluronic acid (HA), and chitosan, or synthetic, such as PVA and polyethylene glycol [87]. Fibrous scaffolds containing collagen combined with drug molecules (Integra^®^, Biobrane^®^, and Terudermis^®^) are commercially available [89,90]. Recently, Bhowmick et al. electrospun a chemical combination of modified HA and chondroitin sulfate to be applied in skin regeneration and proliferation [91]. In addition, alginate-based thin films were synthesized to be applied in implantation for injured and burned skin [92,93].

#### 4.1.4. Oral Tissues

Oral tissue offers a critical function in humans. Dental periodontitis and caries, along with other factors, may lead to damage of the oral tissues [94]. Their wear and tear may result from physical changes while functioning in the environment. The materials inside the oral cavity may be corroded due to exposure to body fluids in the mouth and formation of cytotoxic materials [95,96]. In vitro tissue engineering of dental tissue has been achieved with significant outcomes [97].

A tooth structure with enamel and dentin was first regenerated from tooth buds of porcine third molars [98]. These bud cells were added to biodegradable scaffolds and implanted in rats. The tooth structure was attained within 5 to 7 months. Tissue-engineered teeth using porcine tooth buds and a rat model were regenerated by Duailibi et al. [99]. Polyglycolic acid (PGA) and poly(lactic-co-glycolic) acid (PLGA) were used to cultivate the cells in vitro for six days. These scaffolds maintained the growth of mature tooth tissue. Biodegradable polymer scaffolds were used to produce 3D salivary glandular tissue [97]. In addition, HA and PLA composites were applied to construct temporomandibular joints. These composites were implanted with certain cells to produce osteochondral tissue with vascular bones [100].

### 4.2. Dental Resin-Based Composites

Resin-based materials have been applied as restorative systems owing to the demand for aesthetic restorations. Resin-based composites are widely applied in dentistry. However, critical alarms still remain regarding their biocompatibility. Therefore, numerous reports have been conducted to measure the toxicology of these materials [101]. These structures are limited by polymerization shrinkage stress and the presence of unreacted monomers [102]. In recent years, novel polymerization processes have been designed to enhance the restoration behavior of these composites [102].

There are several resin formulation variables such as catalyst type, diluent content, and cure mode. The influence of resin formulation on the mechanical performance and level of conversion of carbon double bonds of dental restorative resins was discussed by Ferracane et al. [103]. The authors reported a clear correlation between higher levels of conversion and improved mechanical behavior. The influence of post-curing and long-term aging of these materials on cytotoxicity were reported by Bouillaguet et al. [104]. The Ariston pHc (pHc means pH control) was initially moderately toxic, and then became highly toxic for 5 weeks before returning to initial degrees. In addition, estrogen-like materials such as bisphenol A (BPA) and bisphenol A dimethacrylate (BAD) in resin-based dental restorative systems were identified by Lewis et al. by using high-pressure liquid chromatography [105]. The water sorption and solubility of various resin-based restorative dental composites have also been discussed [106]. It was realized that the generic type of materials in resin matrix compositions affected the water sorption and solubility.

### 4.3. Wound Dressing

Many patients are suffering from skin tissue-related devastating trauma. For effective trauma treatment, many studies were achieved to tackle blood loss, physical protection, tissue preservation, and infection [107,108]. Wound healing is a molecular and cellular behavior that provides necessary characteristics to bypass microbial infections [109,110]. Growth factor-secreting cells play a vital role to control the wound healing process [111]. In ideal wound healing processes, the fibroblasts transfer to the wound location to fabricate collagen fibers of the extracellular matrix [112]. In addition, the fibroblasts induce the survival and proliferation of keratinocytes [113]. Wound dressings must accelerate the healing process and offer basic protection for the wound location as well [114,115]. Wound dressings are promising materials that comprise a wide class of materials to provide the latter, including foams, hydrofibers (inter alia), hydrocolloids, hydrogels, films, polymers, cotton, and alginate [116,117,118,119,120,121,122,123]. 

The mechanical and thermal characteristics of different polymers can be improved by incorporating kaolin [124,125,126]. Polymeric dressings can be modified and functionalized to display interesting behaviors such as drug release and hemostatic characteristics [127,128]. Of these, multifunctional hemostatic kaolin–polyurethane (PU) foam composites were synthesized by Lundin et al. [129] by incorporating kaolin into mechanically robust PU foam through a one-step preparation. The initial drug release rate and mechanical characteristics were controlled by the kaolin content. At 5 wt % kaolin and less, the composites provided remarkable resilience and good elasticity of more than 140% strain at break. Moreover, foams with 5 and 10 wt % kaolin promoted the hemostatic capabilities. The kaolin content did not influence the absorption behavior of the PU foams. It is noteworthy to mention that these foams can be applied as multifunctional wound dressings as they exhibit different characteristics such as excellent drug release, hemostatic, cytocompatibility, mechanically robust, and absorption properties. Materials such as chitosan (CS), zeolite, alginate, and collagen were used to achieve localized clotting because of their inherent contact hemostatic behavior [130,131,132,133]. Because of the remarkable antibacterial behavior of titanium oxide (TiO_2_) particles, TiO_2_ was incorporated as a reinforcing agent to the scaffolds for supporting cell growth [134]. For instance, Beheraa et al. synthesized a cost-effective and efficient chitosan (CS)/TiO_2_ composite membrane for wound healing as well as developing mouse L929 fibroblast cells’ survival and proliferation [135]. The membranes were formed with excellent porosity, flexibility, crystallinity, and mechanical strength. The composites offered an excellent antibacterial behavior against *Staphylococcus aureus*. An endpoint MTT assay was applied to test the property of the fabricated membranes to enhance the proliferation and cytocompatibility of fibroblast L929 cells. Additionally, the membranes displayed a greater survival and fast proliferation of L929 cells with reduced apoptosis and oxidative stress. 

Banana peel displays promising characteristics such as high mineral concentration, phenolic structure, antioxidant behavior, and antimicrobial performance against yeast, bacteria, and fungi [136,137,138,139]. Recently, Kamel et al. [140] have synthesized unique nanocomposite membranes from banana peel powder (BPP) and chitosan. BPP serves as an ionic crosslinker and reinforcing agent for chitosan. The swelling properties of chitosan were reduced after incorporating BPP. Furthermore, glycerol increased the molar volume of the rotating units and relaxation time and consequently reduced the frequency. The effect of frequency reduction was enhanced by increasing the BPP loading. This dip may be attributed to polymer/filler interaction, which increases the relaxation time and the relaxed units. CS–BPP membrane at 10 wt % BPP exhibited the highest activity and sensitivity towards *Candida albicans*. In recent years, nanocellulose (NC) has been used in wound-care dressings because of its significant mechanical strength, water-holding capacity, conformability, and elasticity [141]. NC particles exhibited a high surface to volume ratio, which was utilized to enhance cell proliferation and migration. However, the NC particles showed poor antimicrobial behavior against pathogenic microorganisms [142,143]. In detail, a promising bio-nanocomposite from NC, poly(vinyl pyrrolidone) (PVP), and chitosan was fabricated by Poonguzhali et al. for in vitro wound dressings [144]. The PVP and chitosan were morphologically miscible and compatible with each other, forming a biocompatible composite with nanocellulose particles via hydrogen bonding between hydroxyl groups of nanocellulose with carbonyl groups of PVP and with amino and hydroxyl groups of chitosan. The results manifested as a significant enhancement in the mechanical and barrier characteristics of PVP/CS/NC films. At 5 wt % NC, the swelling behavior was improved. The tensile strength was increased by 35.6 ± 5.8, 38.4 ± 2.7, and 39.7 ± 6.9 at 3, 5, and 10 wt % NC, respectively. Additionally, blends with 3 and 5 wt % NC offered excellent cytotoxicity, blood compatibility, and remarkable antibacterial performance against gram-negative and gram-positive bacteria.

**Figure 4:**
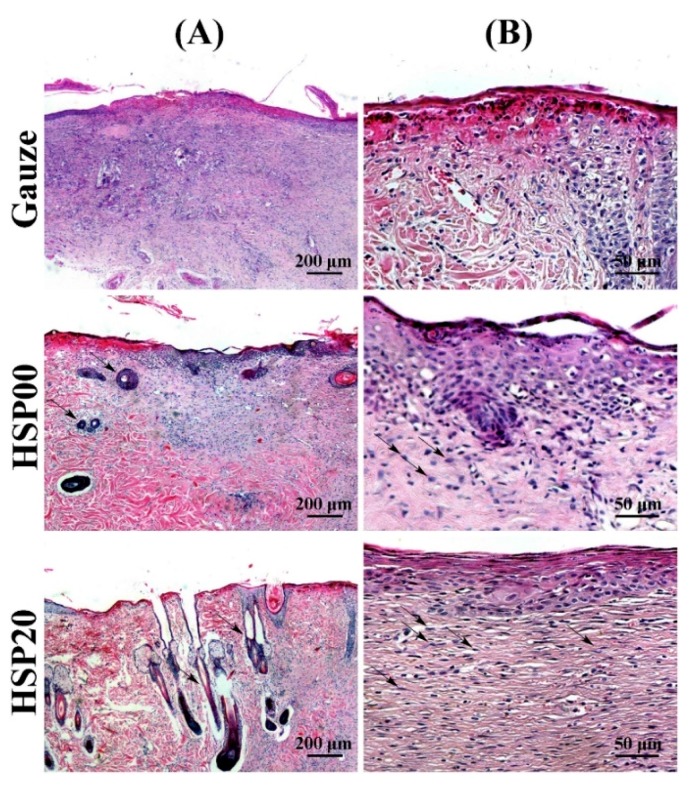
Histological measurement of the skin treated with gauze, HSP00, and HSP20 hydrogel at the 13th day by H&E staining: at (**A**) 50× and (**B**) 200×. Reproduced with permission from [146]. Elsevier, 2017.

Another example of natural materials for wound dressing is silk sericin, which is a natural protein derived from the silkworm with significant properties, such as antibacterial, UV, and oxidation resistance, as well as moisture absorption and release [145]. Yang et al. [146] demonstrated an accelerated wound healing process using silk sericin composites. Authors discussed the potential of a nanoclay lithium magnesium silicate hydrate (LMSH) cross-linked semi-interpenetrating polymer network (semi-IPN) silk sericin/poly((*N*-isopropylacrylamide)(NIPAm)/LMSH) (HSP) nanocomposite hydrogel as a wound dressing. The hydrogels, which were balanced at 37 °C, exhibited a pyknotic morphology with pore sizes ranged from 0.8 to 1.5 µm, while the cross-section structures displayed a transition from a honeycomb to layered morphology, owing to the improved hydrophilicity. With increasing sericin content, water vapor transmission rate (WVTR) values increased by 100 g/(m^2^∙day), while the compression modulus was diminished by 3.859 kPa due to enhancement of the hydrophilic properties. Moreover, the antibacterial behavior was developed after incorporating sericin. Wound healing measurements revealed that the area exposed to a nanocomposite with a mass ratio of 20% (sericin:(sericin+NIPAm)) (HSP20) after 6 days was 83%, three times that of gauze, and was almost recovered after 13 days (Figure 4). This behavior can be attributed to absorption of bacteria by the introduced sericin and adsorption of the decomposed sericin on the surface of bacteria. 

The electrospun fibers exhibit superior properties for application as wound dressings, such as the high surface area to volume ratio and porosity, in addition to having excellent biocompatibility with the structure of the extracellular matrix [147,148,149]. Additionally, fiber composites show semipermeability, absorption, and hemostatic characteristics [150]. According to this concept, thermosensitive poly(*N*-isopropylacrylamide) (PNIPAAm) was electrospun with poly(l-lactic acid-co-ε-caprolactone) (PLCL) and antibiotic ciprofloxacin (CIF) to process a potent wound dressing [151]. The diameter of the processed cylindrical nanofibers was reduced after introducing CIF. The water angle of the prepared mats was temperature-dependent, and the wettability of the PNIPAAm/PLCL nanofibers decreased at a temperature around 32 °C. In vitro drug release measurements revealed that CIF was released from the mats over 200 h. The drug-loaded mats offered excellent proliferation of L929 fibroblasts and biocompatibility as well as remarkable antibacterial behavior against *S. aureus* and *E. coli*. In vivo wound healing studies confirmed the accelerated healing process effected by the drug-loaded nanofibers. Additionally, caffeic acid has two vicinal hydroxyl groups on an aromatic ring, defined as a catechol group [152], and displays different properties, such as antiviral [153], antioxidation [154], antibacterial [155], and anticoagulatory and anti-inflammatory effects [156]. For a wound healing approach, Oh et al. [157] electrospun PCL with chitosan (CS) and chitosan–caffeic acid (CCA) to produce fibers without any bead formation. The average diameters were 1.30 ± 1.07, 1.20 ± 1.22, and 0.94 ± 0.68 µm for PCL, PCL/CS, and PCL/CCA fibers, respectively. In vitro morphology, cell viability, and antimicrobial behavior measurements emphasized that PCL/CCA mats can promote normal human dermal fibroblast-neonatal (NHDF-neo) cell spreading and proliferation with remarkable microbial behavior against *S. aureus* with respect to PCL and PCL/CS fibers. Furthermore, universal tensile machine (UTM) experiments revealed that the tensile characteristics of PCL/CCA fibers were dramatically enlarged compared with PCL and PCL/CS fibers. Although PCL exhibits unique characteristics such as prolonged decomposition rate and good hydrophobicity and crystallinity, PCL is still not bioactive [158,159,160,161]. One of the most common processes achieved to mitigate this drawback is the implementation of bioactive inorganic additives such as bioactive glasses (BGs) [162,163,164,165]. As a case in point, Moura et al. [166] electrospun PCL with BG nanoparticles and silver–cobalt-doped bioactive glass nanoparticles (DB-NPs) to process membranes for wound healing of soft tissue. The weight loss of the fibers was faster after incorporating the nanoparticles. The processed DB-NPs/PCL fibers are considered as promising candidates for soft tissue healing because silver and cobalt ions developed the antibacterial and angiogenic properties of these fibrous membranes. It was found that incorporating the nanoparticles reduced the elongation at fracture and increased the ultimate tensile strength. Considering the tensile characteristics of skin, these fiber composites may be an alternative for wound healing in such a specific tissue as skin. Recently, Wang et al. [167] prepared SF/graphene oxide (GO) nanofibers with a bioinspired nanostructure for wound healing uses. It was realized that graphene oxide improved the antibacterial and biocompatibility behaviors of SF nanofibers [167].

Table 1 represents different polymer-based composites used in various biomedical applications. From Table 1, it is clearly observed that PCL can be employed in many biomedical approaches.

## 5. Conclusions and Future Perspective

Composite materials, with their superior characteristics and diverse applications, are counted as promising materials for research and development. In the context of bioapplications, polymer composites have numerous advantages, such as low cost and using available natural and synthetic matrices, as well as ease and tunable fabrication techniques. Electrospinning, melt-extrusion, solution mixing, latex technology, and in situ processes are the most reported techniques for processing polymer matrix composites. In addition, polymer composites have received great attention due to their effectiveness in tissue engineering, dentistry, and wound healing. Polymer-based scaffolds for tissue engineering applications show high cell adhesion, biocompatibility, biodegradability, and low inflammatory reaction upon implantation. However, polymer composites suffer from some limitations such as releasing acidic byproducts and exhibiting poor cell affinity. Future studies should mitigate these drawbacks by integrating nano-bioceramics with faster degradation rates and incorporating bioactive molecules.

## Figures and Tables

**Table 1:** Summary of different polymer-based composites used in various biomedical applications.

Application		Composite Material	Reference
Tissue engineering	Bone	Poly(lactide-co-glycolide) (PLAGA)/calcium phosphate	Khan et al. [51]
		Bioglass fibers (BGF) (13–93)/PLA	Mehboob et al. [55]
		PLA/Mg rods	Butt et al. [57]
		Polypropylene carbonate (PPC)/poly(d-lactic acid) (PDLA)/tricalcium phosphate (TCP)	Chang et al. [62]
		PLA/ethyl cellulose (EC)/hydroxyapatite (HA)	Mao et al. [60]
		Alginate (AL)/HA/silk fibroin (SF)	Jo et al. [61]
	Blood vessels	PVA/bacterial nanocellulose	Tang et al. [74]
		Chitosan (CS)/gelatin	Badhe et al. [77]
		PLA/CS	Liu et al. [79]
		PCL/gelatin	Jiang et al. [86]
	Skin	Glycoaminoglycoside/collagen	Chua et al. [90]
		Modified HA/chondroitin sulfate	Bhowmick et al. [91]
		AL/ibuprofen	Thu et al. [93]
	Oral tissues	Polyglycolic acid (PGA)/poly(lactic-co-glycolic acid) (PLGA)/porcine tooth buds	Duailibi et al. [99]
		HA/PLA	Schek et al. [100]
Wound dressing		Kaolin/polyurethane	Lundin et al. [129]
		CS/TiO_2_	Behera et al. [135]
		Banana peel powder/CS	Kamel et al. [140]
		Nanocellulose/poly(vinyl pyrrolidone) (PVP)/CS	Poonguzhali et al. [144]
		Nanoclay lithium magnesium silicate hydrate (LMSH) cross-linked semi-interpenetrating polymer network (semi-IPN) silk sericin/poly((*N*-isopropylacrylamide)(NIPAm)/LMSH)	Yang et al. [146]
		Poly(*N*-isopropylacrylamide)(PNIPAAm)/poly(l-lactic acid-co-ε-caprolactone) (PLCL)/antibiotic ciprofloxacin (CIF)	Li et al. [151]
		PCL/CS and PCL/CS–caffeic acid (CCA)	Oh et al. [157]
		PCL/bioactive glass (BG) nanoparticles and PCL/silver–cobalt-doped BG nanoparticles (DB-NPs)	Moura et al. [166]
		SF/graphene oxide (GO)	Wang et al. [167]
